# Macrophages and the development and progression of non-alcoholic fatty liver disease

**DOI:** 10.3389/fimmu.2023.1195699

**Published:** 2023-06-12

**Authors:** Bader Alabdulaali, Fatema Al-rashed, Mohammed Al-Onaizi, Anwar Kandari, Joanna Razafiarison, Dorothy Tonui, Michayla R. Williams, Camille Blériot, Rasheed Ahmad, Fawaz Alzaid

**Affiliations:** ^1^Dasman Diabetes Institute, Kuwait City, Kuwait; ^2^Ministry of Health, Kuwait City, Kuwait; ^3^Department of Anatomy, Faculty of Medicine, Kuwait University, Kuwait City, Kuwait; ^4^INSERM UMR-S1151, CNRS UMR-S8253, Université Paris Cité, Institut Necker Enfants Malades, Paris, France; ^5^Inserm U1015, Gustave Roussy, Villejuif, France

**Keywords:** non-alcoholic fatty liver disease, fibrosis, macrophages, inflammation, Kupffer cells

## Abstract

The liver is the site of first pass metabolism, detoxifying and metabolizing blood arriving from the hepatic portal vein and hepatic artery. It is made up of multiple cell types, including macrophages. These are either *bona fide* tissue-resident Kupffer cells (KC) of embryonic origin, or differentiated from circulating monocytes. KCs are the primary immune cells populating the liver under steady state. Liver macrophages interact with hepatocytes, hepatic stellate cells, and liver sinusoidal endothelial cells to maintain homeostasis, however they are also key contributors to disease progression. Generally tolerogenic, they physiologically phagocytose foreign particles and debris from portal circulation and participate in red blood cell clearance. However as immune cells, they retain the capacity to raise an alarm to recruit other immune cells. Their aberrant function leads to the development of non-alcoholic fatty liver disease (NAFLD). NAFLD refers to a spectrum of conditions ranging from benign steatosis of the liver to steatohepatitis and cirrhosis. In NAFLD, the multiple hit hypothesis proposes that simultaneous influences from the gut and adipose tissue (AT) generate hepatic fat deposition and that inflammation plays a key role in disease progression. KCs initiate the inflammatory response as resident immune effectors, they signal to neighbouring cells and recruit monocytes that differentiated into recruited macrophages *in situ*. Recruited macrophages are central to amplifying the inflammatory response and causing progression of NAFLD to its fibro-inflammatory stages. Given their phagocytic capacity and their being instrumental in maintaining tissue homeostasis, KCs and recruited macrophages are fast-becoming target cell types for therapeutic intervention. We review the literature in the field on the roles of these cells in the development and progression of NAFLD, the characteristics of patients with NAFLD, animal models used in research, as well as the emerging questions. These include the gut-liver-brain axis, which when disrupted can contribute to decline in function, and a discussion on therapeutic strategies that act on the macrophage-inflammatory axis.

## 1 Introduction

### 1.1 Normal physiology and function of the liver

**Figure 1:**
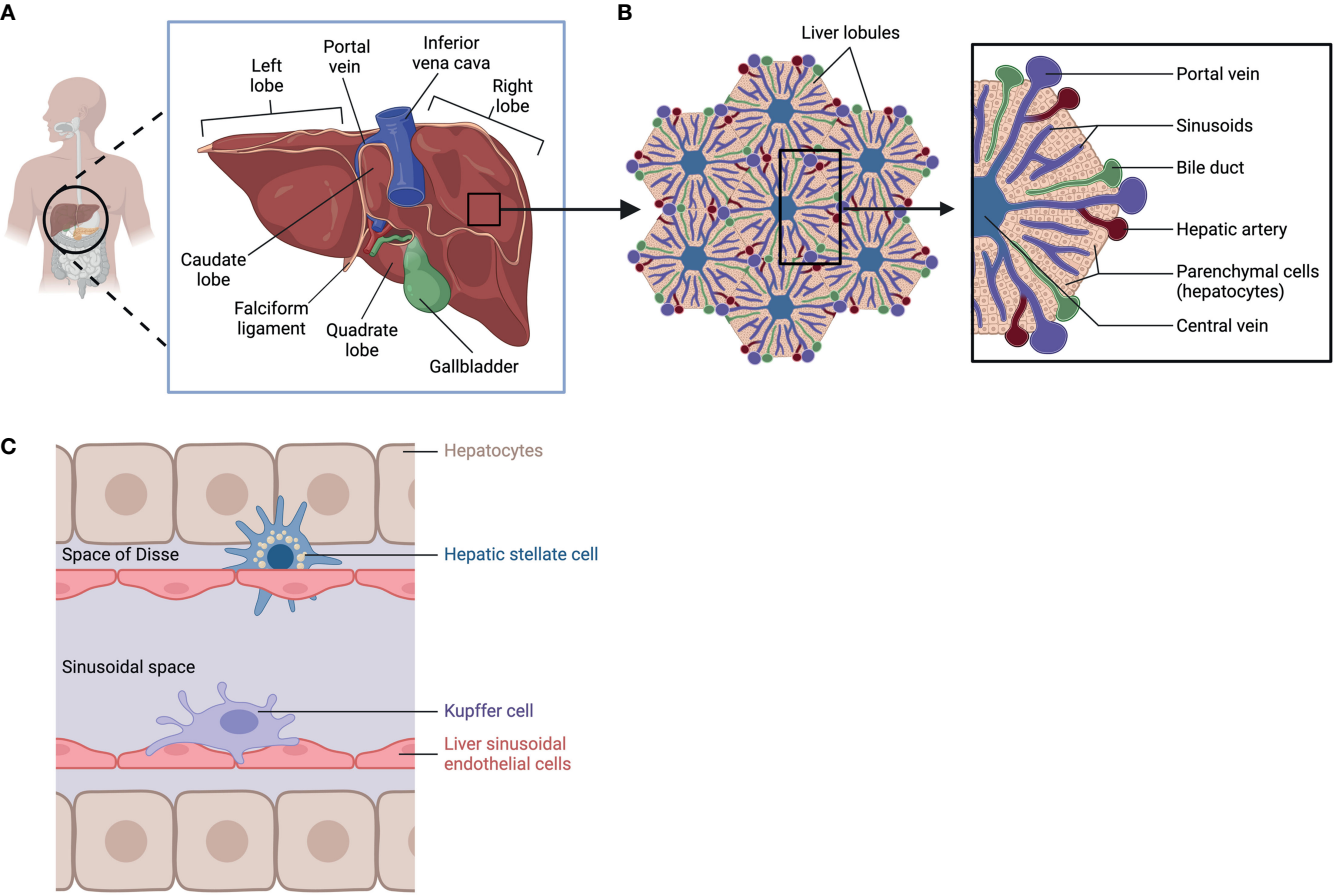
Liver Anatomy, lobular structure, and cell types. **(A)** anatomic location of the liver. **(B)** Lobular structure of the liver. **(C)** Cell types in the liver: hepatocytes, Hepatic stellate cell, Kupffer cell, and Liver sinusoidal endothelial cells. Created with BioRender.com.

Among its many functions, the liver detoxifies and metabolizes components in blood arriving from the hepatic portal vein and hepatic artery, stores glycogen, secretes bile to aid digestion, and produces cholesterol as well as major plasma proteins such as albumin and fibronectin. These synthetic and secretory capacities make the liver the largest gland in the human body (1). Anatomically, the liver is in the upper right abdomen, beneath the right hemidiaphragm, and it is protected by the rib cage. It is separated by visible fissures, the most prominent of which is the umbilical fissure, which is lined by the falciform ligament. Such divisions allow identification of different lobes, for example, the large right lobe and a smaller left lobe (2, 3). The right lobe is further divided into a quadrate lobe and a caudate lobe; these are functional sections where the gallbladder and the inferior vena cava reside (4, 5) (**Figure 1A**).

### 1.2 Tissue architecture and cellular composition of the liver

To carry out its specialized functions, the liver is made up of a variety of cell types organized in lobular structures. One lobule is roughly hexagonal in shape and formed around the central vein with portal triads that demarcate the six corners of each lobule containing bile ducts, sinusoids, branches of the hepatic portal vein, and the hepatic artery (**Figure 1B**). Within its lobular structure are several cell types that cooperate to carry out physiological functions and maintain homeostasis in the tissue microenvironment. The different cell types can be generally divided into parenchymal cells, hepatocytes, and non-parenchymal cells which include hepatic stellate cells (HSC), Kupffer cells (KC), and liver sinusoidal endothelial cells (LSEC) (6) (**Figure 1C**). These cells carry out essential functions in the liver (**Table 1**) and are crucial in responding to injury (7).

**Table 1:** Major cell type distribution in the liver and their known functions.

Cell type	Population frequency (%)	Functions	Location
Hepatocytes	60-80% of total liver cell population	Metabolism of protein, steroids, fats, bile secretion, sugar storage and xenobiotic metabolism.	Hepatic parenchyma
Hepatic stellate cells (HSC)	5-8% of non-parenchymal cells	Storage for fat and vitamin A, control and turnover of extracellular matrix components and secretion of growth factors.	Space of Disse
Kupffer cells (KC) (macrophages)	33.3% of non-parenchymal cells	Role in phagocytosis, cytokines responsible for inflammatory response and liver regeneration (such as TNF-α, IL-1b), prostaglandin E2 (PGE2), antigen processing and iron metabolism.	Sinusoidal lumen
Liver sinusoidal endothelial cells (LSEC)	50% of non-parenchymal cells	Filtration and transport of nutrients from blood, lipid metabolism, adhesion molecules for leukocytes, nitric oxide production, modulating vascular tone, presenting antigens, endocytosis, cytokine secretion, eicosanoid release.	Sinusoidal lining

Table based on (7–9).

Focusing on the immune compartment, KCs are the main immune cells that populate the liver in steady state. They extensively interact with hepatocytes, HSCs, and LSECs. They can stimulate leukocyte chemotaxis and adherence and produce cytokines that influence their activation (10, 11). KCs phagocytose foreign particles and microorganisms in portal circulation coming from the gut (12). These cells are the first line to defense against pathogens, they remove abnormal cells and cellular debris, participate in recycling erythrocytes, and their signaling can dictate inflammation and the immune response (13). KCs also contribute to liver disease when they are dysfunctional (14), this is discussed in more detail below.

### 1.3 Non-alcoholic fatty liver disease

**Figure 2:**
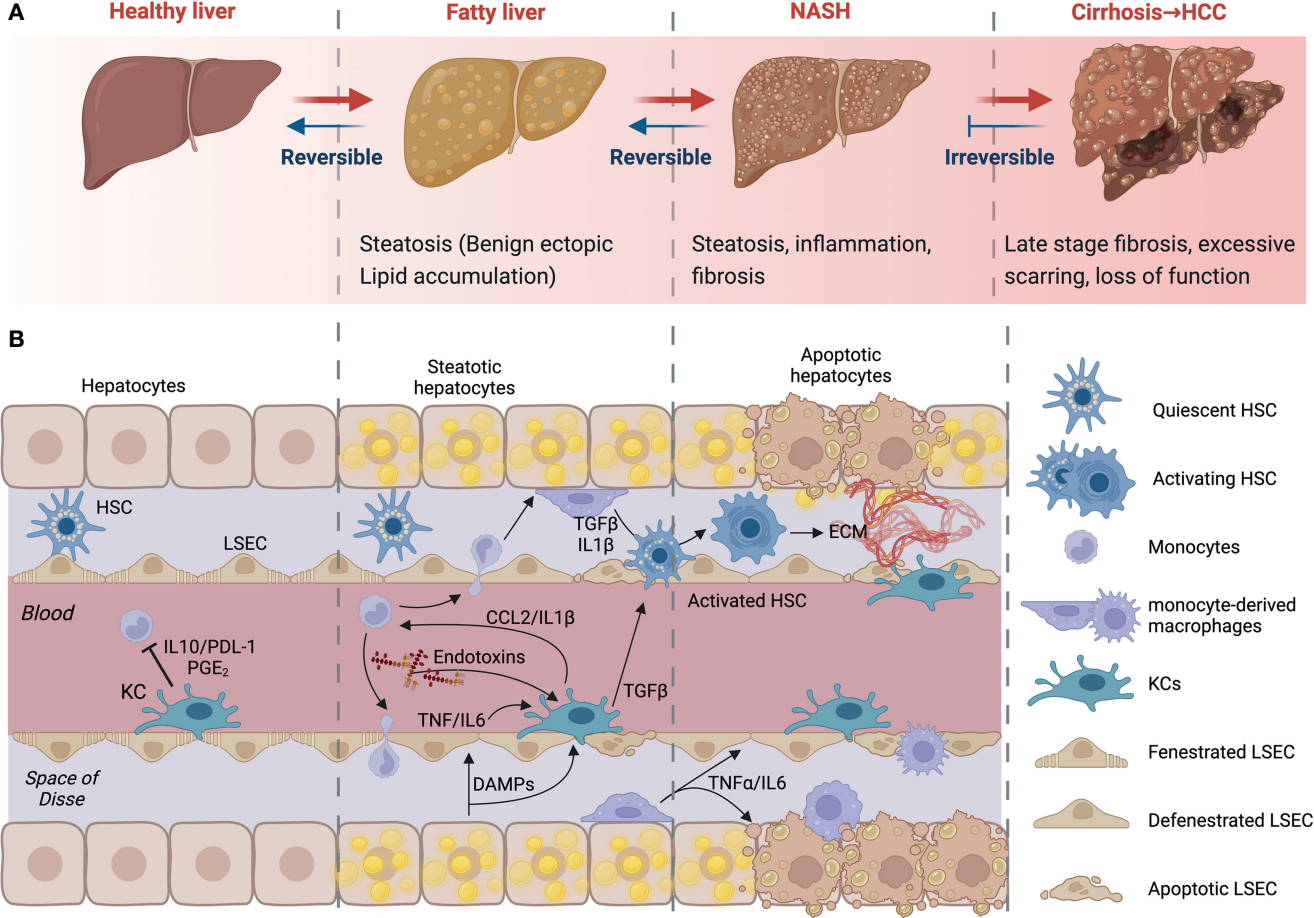
NAFLD progression and cell-to-cell signaling. **(A)** Development and progression of non-alcoholic fatty liver disease (NAFLD) to non-alcoholic steatohepatitis (NASH) and advanced fibrosis. NAFLD can develop into NASH, liver cirrhosis and/or hepatocellular carcinoma **(HCC)** due to several factors. **(B)** Kupffer cells (KCs) recognize imbalances in homeostasis. Their consequent signaling allows the activation of HSCs, contributing to apoptosis and phagocytosis of damaged cells. Early infiltration and differentiation of monocytes contributes to inflammation. KC- and macrophage-derived cytokines induce HSC activation. HSCs will deposit extracellular matrix to create fibrous septa. Created with BioRender.com.

Non-alcoholic fatty liver disease (NAFLD) refers to a spectrum of conditions affecting the liver. These range from benign steatosis to steatohepatitis (NASH), fibrosis, and cirrhosis. Importantly, NASH and fibrosis often occur simultaneously and are the last reversible steps of the condition. At the stage of cirrhosis, liver function is impaired, and patients are at high risk of developing hepatocellular carcinoma (HCC). Liver transplantation is the only possible intervention strategy at or beyond the stage of cirrhosis. Many individuals have the early stages of NAFLD, benign ectopic lipid storage in the form of steatosis, without any signs or symptoms; this is because the presence of fat in the form of simple steatosis does not cause damage to the liver (15–17) (**Figure 2A**). NAFLD and its progressive form NASH have similar risk factors, including overweight or obesity, insulin resistance, high levels of fats, particularly triglycerides, and hyperglycemia, indicating prediabetes or type 2 diabetes (T2D) (17).

The excessive increase of free fatty acids and triglycerides increases lipid oxidation and the production of reactive oxygen species (ROS) and lipotoxicity. This causes cellular damage and the secretion of cytokines, triggering KC activation and the recruitment of monocytes and other immune cells from the circulation. Ensuing hepatocyte apoptosis and HSC activation results in the deposition of extracellular matrix, which can lead to fibrosis if excessive (**Figure 2B**). At this stage, there is obvious persistent scar tissue in the liver and in the blood vessels around the liver. Nonetheless, the liver may still work well, and treating the cause of the inflammation may inhibit further development or even partially reverse the damage (18). However, when the replacement of normal liver tissue by fibrous septa exceeds a certain point, this develops into cirrhosis, the late stage of NAFLD. At this stage, there is extensive scarring and loss of function. Symptoms will include variceal bleeding, ascites, fatigue, and jaundice (19, 20). As a result, the liver stops functioning, urgent medical care and liver transplant surgery may be required.

## 2 NAFLD clinical manifestation and epidemiology

The majority of patients diagnosed with NAFLD are asymptomatic, and this may remain silent until it has developed into cirrhosis (21). Right upper quadrant pain and fatigue are the most noted symptoms among NAFLD patients. Individuals may also have liver fat, based on an imaging assessment or evidence of echogenic liver on ultrasound (22). Serum alanine aminotransferase (ALT) is typically higher than serum aspartate aminotransferase (AST) in liver-related serum tests, indicating hepatocellular stress (23). When advanced fibrosis, cirrhosis, and portal hypertension develop, platelet count gradually declines over time (24).

Globally, almost one billion individuals are diagnosed with NAFLD (25). South America and the Middle East have the highest prevalence, whereas Africa has the lowest (15). In non-obese individuals, the rate is around 10%-30% across Eastern and Western countries (21). There are also differences in gender and ethnic groups in the occurrence of NAFLD in non-obese individuals; for example, the prevalence of NAFLD is much lower in non-obese South Asian women than in non-obese South Asian men (22). Interestingly, NAFLD prevalence in women rises with age, yet does not change with age in men (26). There are several risk factors associated with NAFLD, including primary risk factors related to insulin resistance and metabolic conditions such as T2D, obesity, and dyslipidemia. Secondary risk factors are related to the use of certain medications (corticosteroids, tamoxifen, and amiodarone), metabolically acquired or congenital alterations, surgical operations, and nutritional changes (16). In terms of diagnosis, NAFLD is diagnosed by imaging or by histological assessment of a liver biopsy showing a minimum infiltration of 5% of hepatocytes with steatosis in patients who drink little or no alcohol. Additionally, no other causes of hepatic steatosis are identified, such as Wilson’s disease or drugs such as steroids (27).

## 3 Insulin resistance, liver immunity and inflammation in NAFLD

### 3.1 Insulin resistance

Insulin resistance can in part be attributed to abnormal hepatic insulin processing; this would otherwise physiologically regulate glucose and lipid metabolism. The liver produces glucose using glycogen; this happens with the use of glucogenic precursors in the presence of a high glucagon-to-insulin ratio during a fasting period (28). During the fed state, reductions in glucagon and raised insulin levels signal the liver to raise glucose uptake, cease glucose production, and store the remaining nutrients as glycogen and lipids (29). In pathological conditions of insulin resistance, insulin fails to regulate liver metabolism, resulting in excessive glucose production alongside increased lipid synthesis (30); consequently, NAFLD is associated with insulin-resistance (31).

In obese individuals with T2D and NAFLD, dyslipidemia and hyperinsulinemia are more severe than in individuals without NAFLD (32). Insulin resistance develops in overweight or obese individuals when insulin action on its target tissues is compromised. Excess fatty acids, due to lifestyle factors as well as lipogenesis, accumulate in peripheral tissues such as the liver and AT, which play key roles in lipid storage, synthesis, and metabolism (33). Whilst independently affected by insulin resistance, significant crosstalk also occurs between the liver and AT, this crosstalk has been reported to influence the liver’s immune compartment. Indeed, it has been demonstrated that transplanting donor visceral AT (vAT) from obese mice increased high cholesterol diet (HCD)-induced liver macrophage content, worsening liver damage, compared to mice receiving transplants from lean donors. Adipose tissue macrophage (ATM) depletion prior to vAT transplantation abrogated this effect. On normal chow diets, vAT transplantation increased circulating and hepatic neutrophil numbers in obese-transplanted mice, similarly ATM depletion prior to vAT transplantation reversed this effect (34). Microarray analysis of sorted CD11c+ and CD11c− macrophages isolated from donor adipose tissue showed that obesity increased expression of genes involved in chemotaxis from CD11c+ ATMs. CD11c+ ATMs are known to increase in proportion in obese and insulin resistant conditions (34). These findings indicate that secreted molecules from ATMs act in an endocrine manner and can affect the liver’s immune compartment, influencing susceptibility to, and progression of, NAFLD.

AT also secretes adipokines, a special class of cytokine-like messenger molecules, such as leptin and adiponectin (35). Adiponectin regulates lipid accumulation both in the liver and in AT by inhibiting fatty acid oxidation (36). Moreover, it controls glucose homeostasis through regulating hepatic insulin sensitivity (37). Individuals with NAFLD have lower serum adiponectin levels than the healthy population (38). Hypoadiponectinemia promotes chronic inflammation of the liver that results from dysregulation of fatty acid metabolism associated with insulin resistance (39). Therefore, maintaining the adiponectin level in individuals with NAFLD may prevent progression to NASH. In contrast, leptin levels have been reported to be higher in individuals with NAFLD, and higher levels of circulating leptin are associated with increased disease severity (40). However, *in vivo* studies do not support a causal association since ob/ob mice that lack leptin and develop hyperphagic obesity have severe steatosis (41). Administration of leptin reverses the obese phenotype and improves steatosis. Hence, leptin may play multiple roles in NAFLD (42).

### 3.2 The liver’s immune compartment: macrophages in physiology

KCs are the dominant immune population in the liver; they are *bona fide* resident macrophages that stem from the yolk sac and thus have an embryonic origin (43). KCs are self-renewing and are phenotypically distinct from monocyte-derived macrophages that can infiltrate the liver during disease (44). Monocyte-derived macrophages differentiate *in situ* from circulating monocytes, and thus they originate from bone marrow hematopoiesis (45). Under healthy conditions, rodent models have between 20 and 40 macrophages for every 100 hepatocytes in the liver, making it the organ with the highest proportion of tissue macrophages compared to other tissues. This supports a functional role in homeostasis and normal physiology of the liver (46). They perform a number of tasks, including clearance of metabolic waste and cellular debris (46), regulation of iron homeostasis *via* red blood cell phagocytosis and iron recycling (47), maintenance of cholesterol homeostasis (48), maintenance of immune tolerance (49), and promotion of antimicrobial defense (50).

Once thought to be a homogenous population, recent studies reveal the heterogeneity of liver macrophages, beyond their origin, and evidence continues to accumulate to indicate that certain subpopulations are necessary in maintaining different aspects of homeostasis. Liver macrophage heterogeneity is increasingly deconvoluted with the emergence of precision sorting, single-cell/-nucleus RNA sequencing (scRNA-seq), and macrophage targeting technologies (51). For example, one study demonstrated that there are distinct populations of liver macrophages that function in an inflammatory and non-inflammatory or regulatory manner (52) Another study reported that diet-induced steatohepatitis impairs differentiation of myeloid cells in the liver and bone marrow (53). The diversity and subtypes of liver macrophages are further discussed in a dedicated section below, here we focus on the physiological distinction and roles of liver macrophages.

Interestingly, liver macrophages constitute a separate immune surveillance niche and form a cellular network distinct from those in the hepatic capsule (54). The capsular macrophages are phenotypically and developmentally different from KCs. They arise from circulating monocytes and express the macrophage markers F4/80 and CD64 yet are negative for the canonical resident macrophage markers Clec4F and T-cell immunoglobulin mucin domain containing 4(Tim4) (55). In addition, they can express markers also associated with dendritic cells or M1-like polarization, such as CD11c and MHCII. These cells sense bacteria in the peritoneum and promote neutrophil recruitment to restrict peritoneal bacteria spread to the liver (44). KCs on the other hand are prone to exposure to nutrients and microbial products in the blood rising from portal circulation. KCs encounter this rich venous blood and blood rich in insulin and oxygen from the hepatic artery in sinusoids (56). Mice deficient in KCs have weakened survival following infection with *Listeria monocytogenes*, indicating that KCs plays an important role in the control of bloodborne bacteria. Because of their high exposure to microbial products and nutrient-rich blood, KCs are generally tolerogenic and promote immune tolerance in the liver microenvironment (57). This is achieved by the expression of regulatory or anti-inflammatory molecules like interleukin-10 and prostaglandins, such signaling is important in maintaining immune tolerance and can also promote the differentiation of regulatory T cells (57) (**Figure 2B**).

### 3.3 Liver macrophages in NAFLD development

Although KCs are generally tolerogenic, they retain the capacity to raise an alarm following the detection of danger signals from neighboring cells. KCs will sense disturbances in homeostasis from lipotoxic hepatocytes and endothelial cells; their subsequent signaling places them at the center of an intense cellular crosstalk (**Figure 2B**). This crosstalk allows the recruitment of other immune cells, the activation of HSCs, and the induction of apoptosis and phagocytosis of damaged cells. NAFLD is characterized by an early infiltration of monocytes; these monocytes differentiate into macrophages *in situ* and contribute to the inflammatory response. KC and other macrophage-derived cytokines can target HSCs. TNFα, IL-1β and TGFβ can all induce HSC activation. In turn, HSCs up-regulate several ligands, like CCL2, which are able to attract and regulate the activity of macrophages and other immune cells in NASH (57). Fibrosis progression is largely mediated by liver macrophage-HSC crosstalk (58). Once activated, HSCs will deposit extracellular matrix to create fibrous septa. Thus, macrophages amplify inflammation, support fibrogenic phenotypes and facilitate the survival of HSCs through the chemokines and cytokines they release (57–59). The initial inflammatory hit is key in the progression of the disease (60). KCs can also influence inflammation by binding of bacterial products in the liver’s portal vein. Activation of macrophages is also accompanied by the generation of malondialdehyde because of oxidative stress within the liver. However, KC ablation decreased steatosis and insulin resistance in the liver supporting the notion that KCs can also drive the development of early NAFLD (61). Indeed, an increase in CD68+ cells has been reported in biopsies from patients with NASH compared to those with steatosis (62). In animal studies, high-fat diet (HFD) and methionine-choline-deficient (MCD) diet-fed mice have more liver macrophages that produce pro-inflammatory cytokines (63). In addition, mice fed a MCD diet produced these inflammatory mediators at a higher level after 4 weeks and decreased later, suggesting that the release of these mediators may specifically contribute to progression and that macrophages may play different roles once NASH is established (64). Furthermore, clodronate injections, which deplete phagocytes, decrease steatosis and NASH severity (65). In the absence of KCs, fatty acid oxidation genes and peroxisome proliferator-activated receptor (PPAR) expression were increased in the liver. This mechanism was reported to be dependent on interleukin-1β-mediated suppression of PPAR-α (66, 67). Further, Zhang et al (68) demonstrated that p38α expression is increased in biopsies of patients with NAFLD, relative to control individuals. The importance of macrophage expression of p38α was demonstrated using macrophage-specific knockouts, in which mice deficient for p38α developed less severe liver disease and insulin resistance upon multiple dietary models of NAFLD and NASH. Hence, progression to steatohepatitis is promoted in a p38-dependent manner; p38α promotes macrophage polarization and proinflammatory cytokine release (57).

### 3.4 Macrophage subtyping: origin, heterogeneity, and polarization

**Figure 3:**
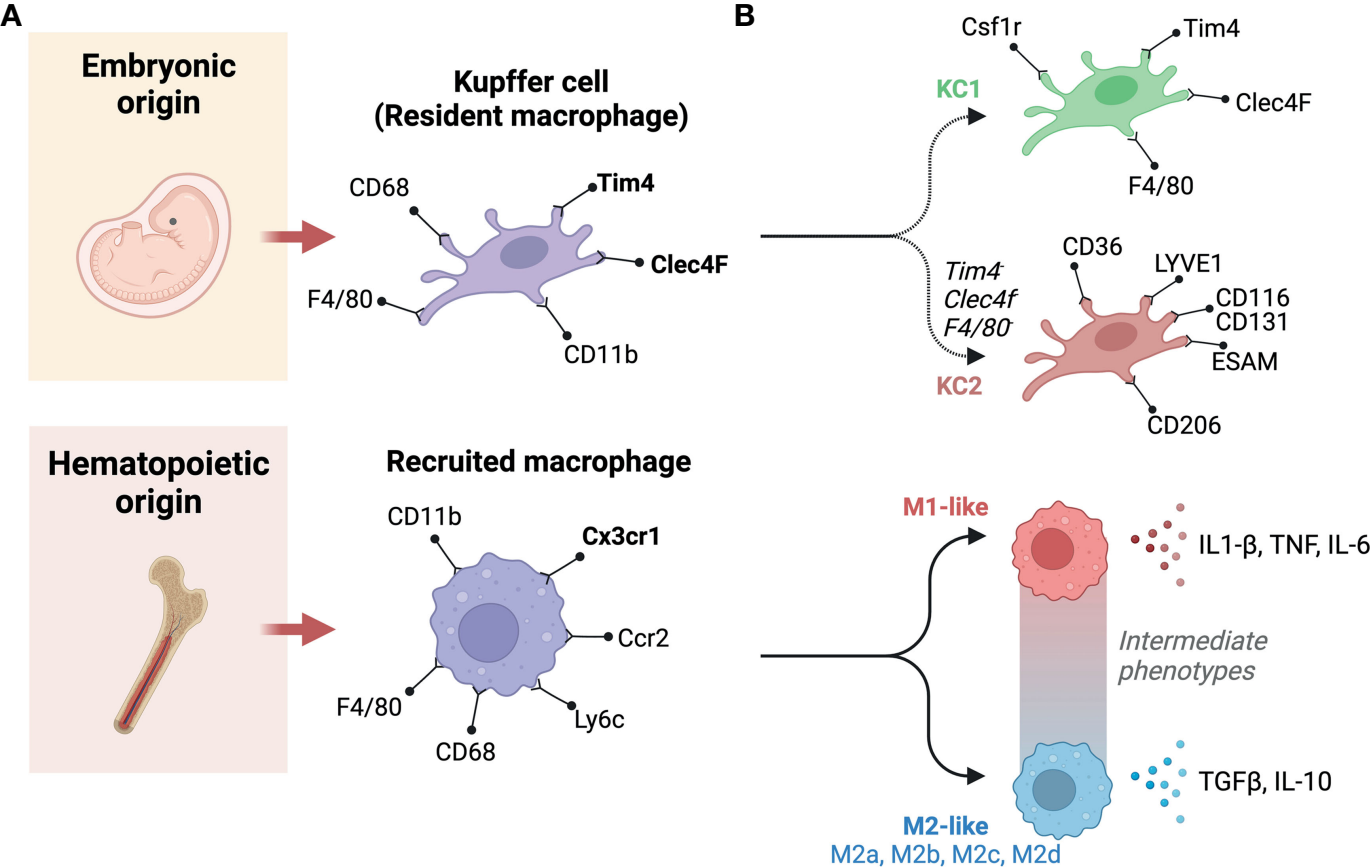
Classification and origins of liver macrophages. **(A)** Based on origin, macrophages can be classified into two subgroups. One originates from the yolk sac (Embryonic origin) and another derives from circulating monocytes that have hematopoietic origins. **(B)** There are two populations of liver resident Kupffer cells (KCs), including KC1s which are CD206 low and Endothelial cell-Selective Adhesion Molecule (ESAM) negative; and KC2s which are CD206high and ESAM+. KC1s express the KC markers: colony-stimulating factor-1 receptor (Csf1r), T-cell immunoglobulin and mucin domain containing 4 (Tim4), C-type lectin domain family 4 member F (Clec4F), and F4/80. In contrast, The KC2 express the markers: CD36, lymphatic vessel endothelial hyaluronan receptor-1 (LYVE1), ESAM, and CD206. Recruited macrophages can be classified based on polarity into M1 and M2. M1 macrophages are canonically inducible by lipopolysaccharide (LPS) and interferon-γ (IFN-γ), whereas interleukin (IL)-4 and IL-13 can induce M2 polarization. M1 macrophages secrete pro-inflammatory cytokines, such as IL-1β and tumor necrosis factor-α (TNF-α). Whereas, M2 macrophages primarily produce anti-inflammatory factors, such as IL-10 and transforming growth factor-β (TGF-β). In between these extremes are a number of intermediate phenotypes, and M2 macrophages can be further categorized into M2a, M2b, M2c, and M2d subtypes. Created with BioRender.com.

Liver macrophages can be categorized in multiple ways, for example, based on their origin, their polarization states, their functional specificities, or the expression of phenotypic markers. These ways of categorizing liver macrophages are a subject of continuous debate, with new categories being continuously proposed as we learn more about population and subpopulation specificities. When origin is used, broadly speaking, liver macrophages are grouped into resident and recruited cells, resident cells being KCs, that are embryonically derived, and recruited macrophages differentiate from monocytes that originate from bone marrow hematopoiesis (69) (**Figure 3A**). In mice, KCs are distinguishable by their variable expression of typical macrophage markers as F4/80^hi^, CD68+, and CD11b^int^ cells, alongside specific liver resident markers Tim4 and Clec4F (70). Recruited macrophages will not carry the latter to markers, and their monocyte progenitors are Cx3cr1+, CD11b+, Ly6c+, and CC-chemokine receptor 2 (CCR2+) (43). CCR2 in particular plays an important role as it regulates the recruitment of monocytes and macrophages. Genetic deficiency of Ccr2 in mice fed a HFD reduced their food intake and decreased their development of obesity compared to wild-type mice. Ccr2 deficiency led to a decrease in AT macrophage content and inflammatory profiles, a substantial rise in adiponectin expression, decreased severity of hepatic steatosis and improved insulin sensitivity in obese mice. An antagonist of CCR2 was found to have significant effects in enhancing insulin sensitivity and lowering macrophages number in adipose tissue in obese mice. In obesity and its related metabolic consequences, CCR2 plays a role in recruiting monocytes to tissues under metabolic stress, sustaining and amplifying inflammation in AT and the liver, and promoting insulin resistance (71). Polarization state can also be used to classify macrophages, it refers to a final stage of differentiation that macrophages reach in response to signals from their immediate environment. Such signals include cytokines, growth factors, exosomes, fatty acids, or various PAMPs and DAMPs. Historically, macrophage polarization states were designated as either M1 or M2 (72). This M1-M2 classification is now considered outdated, macrophages in-fact exhibit enormous diversity, they can adopt intermediate phenotypes that lay on a sliding scale, where M1 and M2 are extremes (**Figure 3B**). When considering phenotypic extremes, M1 macrophages are proinflammatory, releasing an elevated level of proinflammatory cytokines and generating reactive oxygen species, which stimulate inflammatory responses (57, 59). M1 macrophages are activated by GM-CSF, TNF-α, and IFN-γ (73) and are involved in triggering the Th1 response *via* the production of cytokines, including TNF-α (74). They are fueled *via* glycolysis, which has been directly linked to IL-1β production (75). On the other hand, M2 macrophages express molecules that play anti-inflammatory and reparative roles (76). Furthermore, M2 macrophages utilize oxidative metabolism to fuel their functions (75). M2 macrophages are induced by IL-13 or IL-4 (77). They produce polyamines, ornithine, and arginase-1 and induce a Th2 response, promoting immune tolerance and tissue repair (78). M2 macrophages have been found to be protective in the context of inflammatory and metabolic disease, such as insulin resistance; however, they can play deleterious roles in certain conditions with extensive tissue remodeling, such as atherosclerosis and cancer (79). M2 macrophages can be further categorized into four subtypes based on the stimuli and activated transcriptional states: alternatively activated macrophages stimulated by IL-13 or IL-4 (M2a), type 2 macrophages activated *via* IL-10 or IL-1RA (M2b), deactivated macrophages stimulated *via* IL-10 or glucocorticoids (M2c), and M2-like macrophages induced by adenosines or IL-6 (M2d) (79). The canonical transcriptional regulators for M1-like and M2-like polarizations are respectively IRF5, NFkb, STAT1 and IRF4, PPARG, and other members of the STAT and SMAD families of transcription factors (80, 81). In terms of phenotypic identification of these subtypes, both M1-like and M2-like macrophages will express typical macrophage markers (e.g., CD68), in addition to CD11c or CD206 to denote polarization as M1 or M2, respectively (75, 77). Double positivity has been reported for these two markers, as well as associating other functional markers to denote subpopulations (82). Depending on stimulus and context, other markers have been employed to denote M1-like and M2-like polarization. These include CD40, CD86 and high expression of MHC-II for M1-like macrophages, and Dectin-1, CD36 and CD163 for M2-like macrophages (76, 83). The most robust phenotyping strategies should include a combination of appropriate surface markers and the use of intracellular markers that indicate function, i.e., transcription factors responsive to the condition studied.

Using the M1-M2 model, not only are the two extremes functionally distinct, they are also distinct in terms of their cellular metabolic profiles (84). The activation of M1 macrophages leads to the induction of aerobic glycolysis that generates ATP and lactate (85, 86). The pentose phosphate pathway (PPP), that branches from the early stages of glycolysis, is also induced under the same conditions, by IFN-γ or LPS, and produces NADPH and substrates for nucleotide synthesis (87). Additionally, the M1 phenotype is regulated by carbohydrate kinase-like protein (CARKL), implicated in the catalysis of sedoheptulose-7-phosphate (88). CARKL is involved in the metabolic control of pro-inflammatory immune responses by causing a redox shift in M1 macrophages. Consequently, stimulation of an M1-like state by LPS inhibits CARKL and leads to generation of NADH and GSH, while the stimulation of an M2-like state positively regulates CARKL (88). In terms of fatty acid oxidation and oxidative metabolism, IL-4 triggers STAT-6, which results in increased mitochondrial respiration (89, 90). M2 activation facilitates the entrance of pyruvate into the Krebs cycle, supporting the Electron Transport Chain (ETC) and providing the energy required for the remodeling and repair of tissues (84). The activation of M1 macrophages results in increased glycolysis, which aids in microbicidal activity and the management of hypoxia that occurs within the microenvironment of the tissue (91). Glucose, in the cytosol, is converted into L-lactate by glycolysis, in which hexokinase (HK), 6-phosphofructokinase 1 (PFK1) and pyruvate kinase (PK) are key enzymes. During glycolysis, glucose-6-phosphate can be propelled to PPP pathway promoting pentose NADPH and phosphate production. Pentose phosphates are used for the synthesis of amnio acid and nucleotide, while NADPH contributes to the production of ROS and NO (92, 93). Pyruvate is stimulated to lactate in hypoxic conditions, while decarboxylated into acetyl-CoA in the mitochondria in normoxic conditions. Here, acetyl-CoA goes into the TCA cycle, bringing reducing agents to the ETC to generate energy. The TCA metabolite, Citrate, participates in fatty acid synthesis when exported to the cytoplasm, this process can support membrane such functions as membrane synthesis (94). Two breakpoints in M1 macrophages cause the generation of itaconate and the increase of succinate, decreasing pH and stabilizing HIF-1a. HIF-1a results in upregulation of glycolysis and M1-like functions *via* GLUT1 expression and IL-1b production (84). What’s more, the production of NO inhibits the ETC. Nonetheless, M2 macrophages can acquire sufficient ATP from the ETC and *via* the TCA cycle (95).

Beyond the above standard classifications, new subpopulations of macrophages are constantly being discovered, they can functionally lay along the M1-M2 spectrum or have significant phenotypic differences from previously described populations. For example, the recently described functionally distinct KC1 and KC2 populations (96) or Trem2+ lipid-associated macrophage (LAM) (97, 98). The more abundant KC1s are identifiable by their low expression of CD206 and are endothelial cell-selective adhesion molecule negative (ESAM-), whereas KC2s highly express CD206, ESAM, CD36 and the lymphatic vessel endothelial hyaluronan receptor-1 (LYVE1) (52). LAMs were first described in adipose tissue, they enhance the degradation and processing of lipids *via* lipoprotein lipase (Lpl), fatty acid transporter Cd36, and fatty acid binding proteins 4 and 5 (Fabp4, Fabp5) (99). LAMs have also been described in other lipid-rich environments such as in atherosclerotic plaques, here LAM expression of Spp1 and Cd9 is correlated with lesion calcification and a downregulation of pro-inflammatory genes (79). With regards to NAFLD, LAMs can localize to fibrotic areas, macrophage aggregates or to hepatic crown-like structures (hCLS) (100, 101), and have been reported to mitigate liver fibrosis (100). *Daemen et al (* 101) revealed a number of LAM specificities, that they express Trem2, Cd63, Cd9 and Gpmnb; and that they are recruited through Ccr2. Their failure to accumulate in the liver of Ccr2-deficient mice increases liver fibrosis upon high-fat, high-sucrose feeding, indicating that they play an important role in tissue remodeling (101). Interestingly, Trem2 that is expressed on LAMs also exists in a soluble form, and levels in plasma reflect recruitment and expansion of LAMs in the liver (98) (**Figure 3B**).

Whilst significant progress has been made in deciphering the heterogeneity of liver macrophages, under physiological and pathophysiological conditions, most of these studies have been carried out in murine models. More recently, several resources have been developed using novel single cell or single nucleus sequencing to translate findings to humans and shed light on conserved subsets and population features in liver macrophages from human biopsies (e.g., www.livercellatlas.org) (102–104). Thanks to these studies that expand the knowledgebase on human liver macrophages, the translatability of findings from murine studies is increasing with time.

## 4 Macrophage lipid processing in NAFLD

The multiple hit hypothesis, which postulates that simultaneous influences from the gut and adipose tissue produce hepatic fat deposition and inflammation (105, 106), has gained widespread acceptance. Monocytes, recruited and resident macrophages are involved in influencing hepatic lipid accumulation and in triggering inflammation that takes place in NAFLD (107, 108). The role of macrophages in lipid processing is an area of active research, where questions address both cellular metabolism of microenvironmental lipids, as well as macrophage efferent signaling that can influence tissue and systemic lipid homeostasis (109, 110). In a study by Rivera et al (110), it was reported that the depletion of KCs abolished fat accumulation in the liver and delayed the development of NASH induced by an MCD diet. This observation goes hand-in-hand with reduced inflammatory burden (110). This observation was found to be associated with a reduction in two receptors: CD36 and the toll-like receptor 2 (TLR2). This observation is further supported by the requirement of TLR2-dependent fatty acid uptake of diacylated lipoproteins by CD36 for lipid trafficking (67, 99, 110, 111). The innate immune signaling system mediated by Toll-like receptors (TLRs) is implicated in the progression of NASH. However, combination of TLR2 and palmitic acid is required for inflammasome activation, which results in NASH progression. To induce NASH in wild-type (WT) and TLR2-deficient mice, a choline-deficient amino acid-defined (CDAA) deficient diet was fed for 22-weeks. After the recipient mice were lethally irradiated, bone marrow transplanted-TLR2 chimerism was generated. WT mice were treated with TLR2 ligands and/or palmitic acid to stimulate their KCs and HSCs. In response to a CDAA diet, WT mice exhibited severe steatohepatitis and liver fibrosis. Conversely, mice lacking TLR2 did not progress to NASH. Although KCs and HSCs both respond to TLR2 ligands, TLR2 bone marrow chimeric mice demonstrated that KCs play a greater role in the TLR2-mediated progression of NASH than HSCs (112). Conversely, the impact of lipid accumulation in KCs is not always an unwanted outcome. Indeed, in work presented by Leroux and colleagues, it was argued that the upregulation of triglyceride storage in KCs from HFD-fed mice is triggered as part of a normal immune response. In HFD-fed mice, lipidomic and RT-qPCR analysis revealed that KCs become lipid-laden and up-regulate lipogenesis genes *Dgat1* and *Scd1 (* 100, 113, 114). This upregulation is associated with a functional adaptation, that is lipid-laden KCs are primed to recruit lymphocytes more efficiently (CD4+ T cells and B cells in their investigation), of note this adaptation is reversible with the inhibition of lipogenesis. In addition, it was discovered that systemic cholesterol levels influence cellular cholesterol homeostasis in lipid-laden KCs (100). During hypercholesterolemia, macrophage cholesterol efflux is impaired, this drives their polarization towards a proinflammatory phenotype (115, 116). The association is strong between dietary cholesterol intake and hepatic cholesterol in the development of NASH in both human and animal models. A study by Bieghs et al (91), showed that the deletion of *Cd36* and macrophage scavenger receptor 1 (Msr1) in low-density lipoprotein (LDL) receptor-deficient (Ldlr-/-) mice attenuates liver inflammation, indicating involvement of the cholesterol uptake pathway (117). This is supported by large scale epidemiological studies, where dietary cholesterol consumption was independently associated with the development of NASH and cirrhosis (118, 119). It has also been shown that the beginning of hypercholesterolemia might affect membrane fluidity, which, in turn, can cause changes in phagocytic capacity, a process known to be disrupted in murine models of NASH (115, 119–121). It is speculated that the KCs scavenged free cholesterol (including crystals), cholesterol esters, and triglycerides from the remaining big lipid droplets of dying hepatocytes. Naturally, KCs will hydrolyze cholesterol esters and TG before oxidizing the liberated fatty acids. However, in the case of free cholesterol, it cannot be further metabolized, causing it to be retained in KCs, leading to transformation into foam cells, stimulating inflammatory and fibrotic pathways that cause NAFLD to progress to NASH. Together, these observations provide insight into the importance of KCs in participating in the underlying mechanism of hepatic lipid accumulation and inflammation.

## 5 Perspectives: murine modelling of NAFLD and steps in replicating the two-hit model

There is a wide range of mouse models for investigating the pathogenesis of NAFLD. Agreed standards in the use of these models would be valuable to enable valid interpretations and comparisons between the many studies being carried out (122). Furthermore, given that the mechanisms for the development of NAFLD in humans are still not fully defined, it follows that it is highly unlikely that the mechanistically ideal mouse model has been developed. An ideal model for this multi-organ, systemic disease would closely replicate the features of the human disease within a predictable time frame while also showing that its use in potential treatment trials is translatable to the human condition. With echoes of Knudson’s two-hit hypothesis for cancer in the early 1970s, James et al (123) proposed a similar concept for NAFLD, with important factors in disease progression being both steatosis and inflammation, and neither alone being sufficient to lead to NAFLD. It is also entirely possible that NAFLD is reached *via* multiple pathways even though the endpoint definitive features of the disease are similar; this also means that multiple mouse models may be valid for studying different aspects of NAFLD. Here we briefly discuss the main approaches and the features recapitulated by different models (**Table 2**). Characteristics of NALFD progression in humans fall into three broad categories: firstly, metabolic syndrome (obesity, hyperglycemia, insulin resistance, T2D); secondly, specific liver features (steatosis, hepatocyte ballooning, lobular inflammation, liver fibrosis); and, thirdly, wider systemic inflammatory disease (including adipose tissue inflammation, intestinal inflammation with intestinal barrier dysfunction, and alterations to gut microbiota) (122). Mouse models of NAFLD used in research to date also fall into three common categories, or combinations thereof: dietary, chemical, and genetic. Notably, most models use the C57Bl6 strain because of the propensity of this strain to develop metabolic and inflammatory disease, and several features of NAFLD compared with other strains. Although, the same amount of fibrosis and severity of NASH do not always spontaneously arise in these mice as occurs in human disease (124). In terms of methodology, it would be most ideal to be able to show disease progression during an ongoing study, at multiple timepoints. There is also no standard agreed-upon pathological progression in terms of liver histology in mice as it relates to human NAFLD, though at least one has been suggested (118, 125). Thus, progress has been made with murine models but the field’s translatability to humans is still under active investigation (126).

**Table 2:** Comparison of different rodent models of NAFLD.

	Obesity	Insulin resistance	Steatosis	Fibrosis	NASH
Dietary models					
MCD	No	Hepatic IR only	Yes	Yes	Yes
CDAA	No	No	Yes	Yes	Yes
HFD	Yes	Yes	Yes	No	No
HFHS (inc. fructose/glucose/sucrose)	Yes	Yes	Yes	Some	Some
Chemical/pharmacological					
STZ + HFD	Yes	Yes	Yes	Yes	Yes
CCL4	No	No	Yes	Yes	Yes
Paracetamol	No	No	Yes	Yes	Yes
Genetic models					
ob/ob mice	Yes	Yes	Yes	No	No
db/db mice	Yes	Yes	Yes	No	No
PTEN knockout mice	No	No	Yes	Yes	Yes
SREBP-1c transgenic mice	Weight loss	Yes	Yes	Yes	Yes
KK-Ay/a mice	Yes	Yes	Yes	No	No
PPAR-α knockout mice	No	No	Yes	No	No
AOX knockout mice	No	No	Yes	No	Yes (transient)
CD36 knockout mice	No	Hepatic IR only	Yes	No	Yes
ApoE knockout mice	No	No	Yes	No	No

### 5.1 Dietary models

Among the dietary models, a common approach has been to use diets deficient in one or more nutrients. The MCD diet has been adopted for some studies and has the advantage of inducing NASH within a few weeks, but it fails to model the human disease in terms of its often-concomitant obesity. Animals on this diet lose weight, but they still develop hepatic insulin resistance (127). A modification of this diet, the choline-deficient-L-amino-acid-defined (CDAA) diet, overcomes the weight loss issue with the MCD diet; though NASH develops later (20-22 weeks), the diet still elicits limited metabolic syndrome-like characteristics compared to NAFLD in humans (128–131). The established atherogenic diet has also been investigated for its usefulness in NAFLD mouse models: the diet induces steatosis, inflammation, and other histological features of NASH and NAFLD in the mouse liver but does not cause weight gain or insulin resistance (132, 133). Also, simply adding fructose to water was found to result in steatosis, increased weight, and hyperglycemia, though no definitive NASH (134). More commonly used currently are high-fat diets (HFD), which can vary widely in the amounts and types of fat used. These generally induce steatosis and inflammation to a lesser degree than in mice fed an MCD diet. However, they have the advantage of inducing a metabolic syndrome-like pathology that is more similar in profile to human NAFLD, with obesity, insulin resistance, and hyperglycemia (135). Modifications of the HFD, including a combination with fructose (High Fat High Fructose Diet) or sucrose (High Fat High Sucrose Diet) added, either to water or food, result in a phenotype more closely resembling human NAFLD, but not the full extent of liver damage (136, 137). Mice on other HFD variations, the High Fat High Cholesterol Diet (111) and the American Lifestyle Induced Obesity Syndrome (ALIOS) diet which is rich in trans fats (138), also showed additional NAFLD compared with mice on a typical HFD. A large disparity exists between individuals in terms of evolution from NAFLD to NASH, and between mouse strains used for *in vivo* modelling. In one study, an MCD diet was used to induce NASH in C57Bl6/J and Balb/c mice, which exhibit very different responses in terms of innate and adaptive immune responses.C57Bl6/J mice that are more prone to Th1 and M1-like responses, showed more steatosis and lobular inflammation following 4 weeks on the MCD diet than Balb/c mice, which are more prone to Th2 and M2-like responses. Accordingly, neither the Th1/Th2 bias nor IL-4 (interleukin 4) or GATA-3 expression in the liver of either strain is significantly modified by MCD feeding (indicating that innate immune polarization plays a more important role). As a result of MCD feeding, liver mRNA expression of macrophage activation markers M1 (iNOS), MLD (inducible NO synthase), and MCD (CXC chemokine ligand 10) were significantly higher in C57BL6/J mice, however, M2 polarization markers IL-10 and MGL-1 (macrophage galactose-type C-type lectin-1) remained the same. In addition, C57Bl6/J mice fed MCD had a higher level of circulating IL-12 than Balb/c mice fed MCD. Compared with mice fed MCD, macrophages isolated from the livers of C57Bl6/J mice expressed significantly more M1 markers (139).

As well as dietary fat, the form, content and delivery of sugar can be manipulated to influence susceptibility to NAFLD and a NASH-like phenotypes in rodents (140). The considerations have been whether sugar should be delivered in the form of glucose, fructose or sucrose (or in combination), and whether this is provided as part of the solid diet or in drinking water. There are mechanistic advantages and practical considerations for each of these options, for example quantity consumed or calories per gram are easier to control when incorporated into solid food. Fructose alone making up to 60% (w/v) of drinking water can reliably induce steatosis, when fructose is combined with glucose in a solid diet (30% w/v of each), this also results in steatosis (140). Interestingly, when prolonged the 60% (w/v) fructose in drinking water can induce fibrosis, and the same concentration in a solid diet (kcal/w) can induce inflammation and fibrosis. Up to 50% (w/v) sucrose in drinking water, can induce inflammation and fibrosis. In studies of these diet, fructose-supplemented drinking water causes steatosis after 8 weeks, and leads to a significant rise in body weight, and glucose and plasma triglyceride levels (141). Moreover, intestinal bacterial overgrowth is detected after 8 weeks of treatment along with high levels of endotoxin in the portal blood and activation of KCs (134). However, weight gain, followed by development of fat deposits in the abdomen, is not essentially prognostic of steatosis. It has been revealed that fructose causes greater fat accumulation in the liver than sucrose and glucose, despite of the weight gain from glucose and sucrose (142).

### 5.2 Chemical/pharmacological models

Among the chemical mouse models of NAFLD is the well-used streptozotocin induction of T2D. A low dose of intraperitoneal or subcutaneous streptozotocin shortly after birth leads to inflammation and the destruction of pancreatic islets; when combined with a HFD, animals progress quickly through steatosis, NASH, and increased fibrosis to hepatocellular carcinoma (HCC) around 20 weeks of age (143). The use of carbon tetrachloride (CCl_4_) in multiple IP doses leads to significant but reversible fibrosis (144); when combined with HFD steatosis, NASH and increasing fibrosis are seen (145);however, the systemic metabolic aspects of NAFLD are absent (146). One-time administration of diethylnitrosamine (DEN) has been used to model HCC; when combined with a HFD, animals do gain weight and develop NASH, as well as some other aspects of the metabolic syndrome associated with NAFLD (147). In each case, the value of toxicity or drug-induced NASH is questionable because it most likely doesn’t parallel the progression of the disease in humans and the mechanisms leading to fibrogenesis may be completely independent.

### 5.3 Genetic models

Leptin is an adipokine that centrally controls appetite. The leptin knockout mice, Lepob/Lepob (ob/ob), are obese, insulin resistant, and hyperglycemic, and show some degree of steatosis. Likewise, leptin receptor knockout mice, Leprdb/Leprdb (db/db), have a similar phenotype to ob/ob, and in both cases, an additional stimulus is required for the development of NASH. Several groups have explored the combination of genetic models with dietary or chemical stimulus to induce NAFLD with varying degrees of success (148–151). Mice with a mutation in Alms1, which encodes a protein involved in control of satiety *via* the hypothalamus, also require a dietary stimulus (HFD) to induce symptoms similar to NAFLD, with the lipid profile not mirroring the human disease (152). Sterol regulatory element-binding protein (SREBP-1c) transgenic mice also develop insulin resistance and steatosis (123). Better for examining the metabolic syndrome are the established atherosclerosis models such as ApoE-/- mice (apolipoprotein E deficient) and Ldlr-/- mice (LDL receptor deficient); these animals are predisposed to hypercholesterolemia, atherosclerosis, and obesity, and on a HFD or Western diet will also develop NAFLD/NASH (153).

## 6 Perspectives: emerging roles of the gut-liver-brain axis

Gut-liver axis refers to the relationship between the gut and liver at the proximal anatomical and physiological level. Bile acids (BA) produced from the liver are secreted into the duodenum to aid in lipid metabolism and bacterial homeostasis. Products of ileal absorption are then transported back to the liver *via* portal circulation. As such, the liver and the gut are always in contact with each other. Playing an important role in regulating this contact are the KCs and hepatic dendritic cells (DCs) that line sinusoids to filter and remove gut-derived microorganisms, microbial products and microbe-associated molecular patterns (MAMPs) from portal circulation (154).

Similar to the liver, the intestine has its own set of macrophages, mainly in the lamina propria (LP). Intestinal macrophages are most abundant in the colon due to the higher microbial load. Macrophages are constantly replenished in the LP because of extravasated Ly6C^lo^ CX3CR1^hi^ MHCII^+^ monocytes that develop into mature Ly6C^lo^ CX3CR1^int^ macrophages. LP macrophages are highly phagocytic and typically remain unresponsive towards harmless bacteria and food antigens. They play an important role in the induction of oral tolerance by sampling the gut lumen and presenting antigens to DCs during the induction process. Related self-renewing macrophage are also found near intestinal neurons and blood vessels. Under the LP, macrophages associated with neurons express genes typically enriched in microglia, the specialized central nervous system (CNS) macrophages. As a result, these genes may be essential for maintaining a healthy local neuronal population in a manner similar to microglia in the CNS (154).

**Figure 4:**
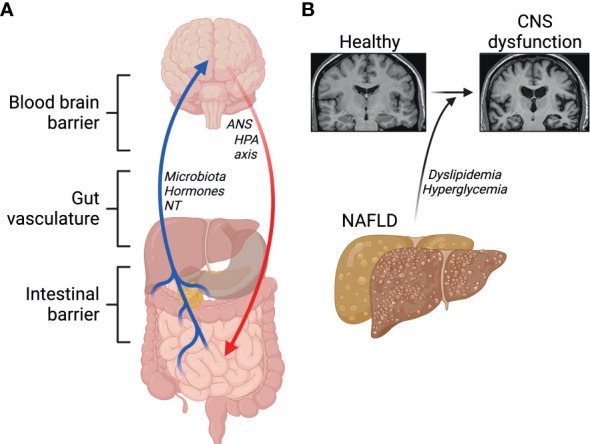
Emerging roles of the gut-liver-brain axis. **(A)** Barriers and signals between the gut, the liver and the brain. NT, neurotransmitters; ANS, autonomic nervous system; HPA, hypothalamic-pituitary-adrenal. **(B)** NAFLD and its comorbidities contribute to central nervous system (CNS) dysfunction. Created with BioRender.com.

The contact of peripheral macrophages with neurons has led to a growing field of research aiming to elucidate the effects of the gut-liver-brain axis in health and disease. The gut-liver-brain axis is complex with several regulators such as the intestinal barrier, gut-vasculature barrier, blood-brain barrier, and the immune system. The gut-brain axis is involved in modulating several physiological and homeostatic functions (155, 156). Namely, the CNS regulates gut function through the hypothalamic-pituitary-adrenal axis (HPA) and the autonomic nervous system. Moreover, the gut also alters CNS function through microbiota-derived molecules, gut hormones, and neurotransmitters (**Figure 4A**). These molecules enter the CNS *via* the enteric nervous system, vagus nerve, or the immune system.

Gut-liver interaction is regulated by enterohepatic circulation, and a disruption of this axis has been suggested to contribute to NAFLD, which itself has been associated with lower cognitive performance. This suggests a link between abnormal liver metabolism and neurodegenerative diseases, including Alzheimer’s disease (AD) (157–159). Physiologically, circulating Amyloid β peptides (Aβ) are detoxified by the liver. A key feature in AD is the accumulation of Aβ plaques and defective clearance by the liver through lipoprotein receptor-related protein 1 (LRP1) (160). In healthy conditions, insulin promotes LRP1 translocation to the cell membrane in hepatocytes favoring Aβ clearance (161). Low hepatic expression of LRP1 is observed in patients with liver diseases, accompanied with high levels of circulating Aβ, suggesting impaired LRP1-mediated clearance of Aβ (160). Insulin resistance impairs LRP1 translocation, contributing to its functional impairment in clearing Aβ (161). In one study by Więckowska-Gacek et al. (162), long-term feeding of Western diet in mutant amyloid protein precursor (APP) AD mice, which express mutant human APP with the Swedish mutation, accelerated the rate of several abnormalities in the brain, compared to normal diet fed APP AD mice. Interestingly, in the same study, liver damage in Western diet fed mice was correlated with the deposition of Aβ in the brain (162). Indeed, evidence shows that diets high in simple sugars, salt, cholesterol, saturated fatty acids, trans fatty acids and low in fiber and mono- and polyunsaturated fatty acids, as in the Western diet, impair Aβ clearance in the periphery by the liver through increased activity of the receptor for advanced glycation end-products (RAGE) (8, 159, 163). Moreover, mice fed a Western diet exhibit impairments in the blood-brain-barrier (BBB), which has been shown to trigger oxidative stress, leading to enhanced activity of β-secretase and γ-secretase, which promote the generation of Aβ peptides (9, 162). Hypercholesterolemia has also been proposed to play a major role in the progression of neurodegenerative diseases (164) (**Figure 4B**). Elevated levels of cytochrome P450 CYP27A1 and CYP7A1 isoforms in hepatocytes during NAFLD underlie the conversion of serum cholesterol into 27-hydroxycholesterol, which crosses freely through the BBB (116, 165, 166). High levels of 27-hydroxycholesterol in the brain leads to oxidative stress and the development of AD pathology (165, 167–169). Both dyslipidemia and the systemic inflammation, that often follows, are major candidate contributors to neuroinflammation and compromised cognitive function.

Evidence also shows that the hepatic branch of the vagus nerve, which serves as a major connection between the liver and CNS, plays a pivotal role in the progression of liver cirrhosis, and influences the gut microbiome (145, 146). Another key player in the liver-brain axis is the brain-derived neurotrophic factor (BDNF) (170, 171). In the brain, BDNF plays several roles including, but not limited to regulating synaptic plasticity, neuroinflammation, and neurogenesis (172–175). Furthermore, BDNF can also modulate insulin signaling and liver disease in animal models of cirrhosis and alcohol-induced liver disease (176–178). Interestingly, experiments show that mice with cirrhosis have high levels of the BDNF in the liver, and hepatic vagotomy levels were found to be significantly reduced in the liver but increased in the brain. While these studies clearly indicate that BDNF plays a role in liver and brain diseases, further studies are warranted to elucidate its downstream effects in the CNS and periphery. A common mechanism leading to disruption at the gut, liver or brain levels is inflammation. This inflammation is caused by macrophages that reside in each compartment. The most plausible starting points for dysregulation of the gut-liver-brain-axis are either at the level of the intestine or in the liver itself. These two tissues are populated by specialized macrophages very susceptible to encountering bacteria or bacterial products, they are also strongly affected by metabolic stress and insulin resistance at a very early stage. Subsequent inflammatory signaling may act in an endocrine manner, similar to crosstalk from AT, to influence microglia in the CNS or induce hyper-responsiveness of circulating monocytes, making them more likely to cross the BBB and contribute to neuroinflammation. Other potential mechanisms linking these systems is microbiome-mediated signaling *via* bacterial metabolites or other products, that can act at the level of each organ; or actions of nerve-associated macrophages (NAM). The study of peripheral NAMs is in its infancy, they have been identified in a limited number of organs and have been attributed physiological roles in few conditions. One relevant example is the role for NAMs in adipose tissue, these have been described to regulate catecholamine release from sympathetic nerves that populate the tissue. Nerve-interactive macrophages have also been identified in the liver and are known to induce neuropathy and promote insulin resistance, however their mechanisms of activation remain unknown (179). In the context of alcohol-associated liver disease however, KCs have been demonstrated to respond to gut-derived catecholamines with hepatoprotective outcomes (180).

## 7 Concluding remarks, therapeutic and technological innovations in NAFLD

NAFLD has become one of the most common liver diseases worldwide. Its complicated pathogenesis is related to disorders such as obesity, insulin resistance, T2D, and hypertension. All these conditions carry the risk of initiating or aggravating the development of steatosis, fibrosis, cirrhosis, and hepatocellular carcinoma. Oxidative stress and changes in chemokines, adipokines, and anti- or pro-inflammatory cytokines are key factors in the development of NAFLD. KCs and monocyte-derived macrophages are phagocytes in the liver that execute several metabolic and immune-related functions under homeostasis and in disease. Macrophages play a vital role at all stages of NAFLD and contribute to pathology. Therefore, targeting liver macrophages is promising as a therapeutic avenue. Focusing on macrophages to treat NAFLD in its early stages may be an ideal method to reduce the damage or likelihood of progressing to the later stages of NASH. Also, further interpretation of the crosstalk between macrophages and other immune cells similarly remains a promising area of exploration, as it will not only improve our understanding of NAFLD pathogenesis but also help us discover novel circulating biomarkers or therapeutic interventions for NAFLD (181). As such, additional studies are required to further investigate the function of specific macrophage subpopulations and their specific markers or receptors in NAFLD progression and target them for treatment (182).

Regulating macrophage subpopulation abundance, or their specific functions, is an area of active research. To meet this research need, in-depth mechanistic studies at the transcriptional or epigenetic levels must be carried out. Epigenetic regulation plays an important role in dictating macrophage fate. Targeting transcriptional machinery, *via* transcription factor activity or epigenetic modifications is another encouraging strategy (183). Carotenoids, for example, are also promising anti-inflammatory and antioxidant molecules; in preclinical studies, they impede inflammation, steatosis, and fibrosis. However, there is no clinical evidence to date that carotenoids have beneficial effects against NAFLD in patients. Further studies are warranted to demonstrate the potential role of carotenoids, or carotenoid-related molecules, in the prevention and treatment of NAFLD (184).

Inflammation and fibrosis resulting from NASH have also been associated with galectin-3 proteins. Galectin-3 proteins are members of a family of glycoproteins that bind to galactose-containing oligosaccharides. Their expression on macrophages suppresses the pro-inflammatory phenotype and upregulates expression of type-2 molecules, such as TGFb (185). Pre-clinical outcomes of a galectin-3 inhibitor, the GR-MD-02, indicated its success in reversing NASH with cirrhosis, supporting clinical development trials in future that target advanced fibrosis/cirrhosis with NASH. GR-MD-02 is derived from a natural plant compound that contains galactose residues and binds to galectin-3 (186). Another strategy targeting macrophages has been use of a dual inhibitor of CCR2 and CCR5, Cenicriviroc. Cenicriviroc acts by decreasing recruitment of pro-inflammatory macrophages in mice with liver fibrosis (187). Cenicriviroc was proven effective in a phase II clinical trial (NCT02217475) of NASH patients with liver fibrosis. Fibrosis was ameliorated in patients who were administered 150 mg of Cenicriviroc for 2 years, with a good safety profile (188). However, a phase III trial (NCT03028740) with the same compound, in individuals with advanced fibrosis and cirrhosis, was terminated due to lack of efficacy (189). Therefore, a strategy targeting CCR2/CCR5 may only be effective in early stages of NASH, but not in advanced fibrosis or cirrhosis.

Other targetable molecules include those secreted from macrophages, that may have paracrine or endocrine effects. For example, enhanced production of IP-10 and MCP-1 by the intrahepatic KCs can trigger the development of NASH. In addition, silencing of TNFα in myeloid cells abrogated the production of these chemokines and prevented the development of NASH. Thus, Inhibition of TNFα might delineate a novel therapeutic target in NASH. Therefore, blockade of TNFα might represent a novel therapeutic target in NASH with the potential to limit tissue injury and possibly prevent the progression to severe liver disease (190). With a low-dose dexamethasone conjugate, selective anti-CD163 targeting of KCs inhibited fructose-induced steatohepatitis in rats without systemic side effects. Therefore, CD163 positive macrophages could be a potential therapeutic target to inhibit the progression of further liver damage in NASH patients (191). Targeting specific KC subsets, like the recently identified KC2s may also be an avenue for future intervention. The CD206hi ESAM+ KC2 regulate liver metabolism in the obese murine model of obesity by expressing the fatty acid transporter CD36. Silencing CD36 improves glucose homeostasis and based on a mechanism that improves oxidative stress in KC2 cells. In obesity, the reduction of oxidative stress in KCs has been shown to enhance liver metabolism and reduce ROS levels within the liver. As such, strategies that affect KC2 metabolic function can be considered for the regulation of liver metabolic diseases (52).

Innovative technological developments and studies in appropriate preclinical models will further help elucidate the complex pathophysiology of NAFLD and the roles of macrophages. Further basic and clinical research is essential for better understanding the molecular mechanisms by which the chemokine system mediates hepatic and adipose inflammation, as well as their interaction in the progression of NAFLD (192). To improve the status of patients with NAFLD, combination approaches are required that include lifestyle modifications personally tailored to the patient’s disease drivers (obesity, T2D) as well as specific pharmacological interventions that target influential pathways (193). Furthermore, classifying patients at specific disease stages might provide a more individualized treatment strategy, which could improve treatment responses. In conclusion, combinational treatment approaches could lead to beneficial effects. Specifically, combining anti-inflammatory and metabolic approaches is a promising strategy for future clinical studies (194).

## Author contributions

BA and FAlz contributed to conception and design; BA, JR, DT and FAlz contributed to making figures; BA, FAl-r, MA-O, AK, JR, DT, MW, CB, RA and FAlz contributed to writing the manuscript and wrote sections of the manuscript. All authors contributed to the article and approved the submitted version.
